# COVPRIG robustly predicts the overall survival of IDH wild-type glioblastoma and highlights METTL1^+^ neural-progenitor-like tumor cell in driving unfavorable outcome

**DOI:** 10.1186/s12967-023-04382-2

**Published:** 2023-08-08

**Authors:** Hang Ji, Fang Wang, Zhihui Liu, Yue Li, Haogeng Sun, Anqi Xiao, Huanxin Zhang, Chao You, Shaoshan Hu, Yi Liu

**Affiliations:** 1 Department of Neurosurgery, West China Hospital Sichuan University, No. 37 Guoxue Lane, Chengdu, Sichuan China; 2 Department of Neurosurgery, Zhejiang Provincial People’s Hospital, No. 158 Shangtang Road, Hangzhou, Zhejiang China

**Keywords:** IDH wild-type GBM, Nomogram, Systemic review, Integrated discriminative improvement, Neural progenitor cell-like malignant cell

## Abstract

**Background:**

Accurately predicting the outcome of isocitrate dehydrogenase (IDH) wild-type glioblastoma (GBM) remains hitherto challenging. This study aims to **Co**nstruct and **V**alidate a Robust **Pr**ognostic Model for **I**DH wild-type **G**BM (COVPRIG) for the prediction of overall survival using a novel metric, gene–gene (G × G) interaction, and explore molecular and cellular underpinnings.

**Methods:**

Univariate and multivariate Cox regression of four independent trans-ethnic cohorts containing a total of 800 samples. Prediction efficacy was comprehensively evaluated and compared with previous models by a systematic literature review. The molecular underpinnings of COVPRIG were elucidated by integrated analysis of bulk-tumor and single-cell based datasets.

**Results:**

Using a Cox-ph model-based method, six of the 93,961 G × G interactions were screened to form an optimal combination which, together with age, comprised the COVPRIG model. COVPRIG was designed for RNA-seq and microarray, respectively, and effectively identified patients at high risk of mortality. The predictive performance of COVPRIG was satisfactory, with area under the curve (AUC) ranging from 0.56 (CGGA693, RNA-seq, 6-month survival) to 0.79 (TCGA RNAseq, 18-month survival), which can be further validated by decision curves. Nomograms were constructed for individual risk prediction for RNA-seq and microarray-based cohorts, respectively. Besides, the prognostic significance of COVPRIG was also validated in GBM including the IDH mutant samples. Notably, COVPRIG was comprehensively evaluated and externally validated, and a systemic review disclosed that COVPRIG outperformed current validated models with an integrated discrimination improvement (IDI) of 6–16%. Moreover, integrative bioinformatics analysis predicted an essential role of METTL1^+^ neural-progenitor-like (NPC-like) malignant cell in driving unfavorable outcome.

**Conclusion:**

This study provided a powerful tool for the outcome prediction for IDH wild-type GBM, and preliminary molecular underpinnings for future research.

**Supplementary Information:**

The online version contains supplementary material available at 10.1186/s12967-023-04382-2.

## Introduction

Glioblastoma (GBM) represents the most frequent and devastating brain malignancy. Despite standard therapeutic modalities, the outcome of patients remains dismal [1–3]. Isocitrate dehydrogenase (IDH) gene mutation plays a fundamental role in the carbohydrate metabolism, the tumor microenvironment (TME), and was involved in compromising the anti-tumor immune response [4, 5]. Approximately 90% of GBM is IDH wild-type, which represents the most lethal subtype of glioma [5, 6]. As there lacks effective treatment modalities, it is in urgent need to accurately predict the prognosis of IDH wild-type GBM for individual tailored therapeutic regimens and improvement of clinical management.

The outcome of IDH wild-type GBM can be heterogeneous. IDH wild-type GBM is classified as proneural, classical, and mesenchymal subtypes based on the transcriptome, with the latter having the worst prognosis [4, 7]. Recently, four tumor cell states have been identified using the advanced single-cell RNA sequencing, which was implicated in treatment resistance and prognosis [8]. In addition, O-6-methylguanine-DNA methyltransferase (MGMT) promoter methylation, telomerase reverse transcriptase (TERT) promoter mutations, and N6-methyladenosine-mediated RNA modification were found to be associated with patients’ outcome [9–13]. Nevertheless, the clinical application of these novel biomarkers for prognostic prediction remains limited. Recently, several studies have endeavored to develop prognostic models for IDH wild-type GBM based on multi-omics data [14–20]. Notably, gene–gene (G × G) interactions have profound biological implications. For instance, the prognostic value of HIF1A in non-small cell lung cancer is altered with EGLN2 expression [21], and gene signature associated with T cell dysfunction can be screened by assessing G × G interactions [22]. In addition, G × G interactions can serve as the basis for constructing prognostic models [23, 24].

Compelling evidence illuminates that stem-like tumor cell, which is at the interface of neural and glioma biology, is essential in tumor progression and treatment resistance [25–27]. One step further, neural progenitor cell-like (NPC-like) tumor cells characterized by CDK4 has been defined in GBM [8]. These cells retain the potential to differentiate into other cells and have an enhanced invasive capacity upon neuronal activity induced calcium signals [28, 29]. Despite being an ideal therapeutic target, the inherent cytotoxic reagents resistance of NPC-like tumor cells prompts a deeper understanding of the cancer biology of these cells.

In this study, we developed a robust prognostic model for IDH wild-type GBM through incorporating a novel parameter, G × G interaction [23, 24], to effectively identify patients at high mortality risk. In addition, comprehensive bioinformatics analysis pinpointed a subset of NPC-like cells as an essential player in driving unfavorable outcome.

## Materials and methods

### Data collection and sample pre-procession

The expression profile and corresponding demographic of 800 IDH wild-type GBM from 3 programs were first enrolled for the construction of COVPRIG, including TCGA RNA-seq (RNAseq, n = 143) [30], CGGA693 (RNAseq, n = 190) [31], TCGA microarray (microarray, n = 372) and GSE16011 (microarray, n = 95) [32]. Any sample missing survival information or simultaneously missing age, IDH gene mutation, and MGMT promoter (MGMTp) methylation status was excluded. The RNA-seq profiles were fragments per kilobase of exon model per million mapped fragments (FPKM) normalized, and both RNA-seq and microarray data sets were log-transformed. Three 10 × Genomics-based (GSE131928, GSE138794, GSE139448) and a Smartseq2-based (GSE131928) GBM single-cell expression profiles were employed for exploring cellular dynamics and interactions [8]. To expand the application of COVPRIG, all GBM samples (IDH wild-type and IDH mutant) included in the TCGA RNA-seq, TCGA microarray, CGGA693, GSE16011, GSE4271 [33], and GSE7696 [34] with complete survival information were included (nsample = 956) for assessing the prognostic significance of the model. All-cause death was defined as the outcome, which was curated by each program. Patients were followed for a period of at least 2 years’ post-surgery or until death. Demographics of samples were curated in Additional file 7: Tables S1 and S2. Gene signatures, including NABA core matrisome, NABA extracellular matrix (ECM) affiliated, NABA matrisome associated, Reactome ECM organization, and HALLMARK interferon alpha response was collected from the MSigDB [35] (https://www.gsea-msigdb.org/gsea/msigdb).

### Gene main effects, G × G interactions selection and COVPRIG construction

#### Gene main effects selection

A total of 9594 common mRNAs from TCGA RNA-seq, TCGA microarray, CGGA693, and GSE16011 cohorts were included. Individual genes were enrolled into the Cox-ph model (Model 1) to determine their independent prognostic significance. Given the potential differences between RNA-seq and microarray data [36], gene main effects of significant coefficients in the TCGA RNA-seq and TCGA microarray were intersected. Then, candidates were further screened using multivariate Cox regression, with age being the covariate.

$$\mathrm{Model} \,1{:}\, h(t)={h}_{0}(t)exp({beta}_{gene i} \times {gene}_{i} +{beta}_{age} \times age)$$

#### G × G interactions selection

The prognostic significance of G × G interactions was determined based on Model 2. To ensure the potential significance while incorporating an appropriate number of variables, we calculated the median absolute deviation (MAD) was calculated for each gene. The top 1200 highly variable genes in TCGA RNA-seq, TCGA microarray, CGGA693, and GSE16011 were intersected, resulting in 434 common genes, which were randomly paired into 93,961 G × G interactions. Likewise, G × G interactions of prognostic significance in both the TCGA RNA-seq and microarray were intersected.

$$\mathrm{Model}\, 2{:}\, h(t)={h}_{0}(t)exp({beta}_{gene i} \times {gene}_{i} +{beta}_{gene j}\times {gene}_{j}+{beta}_{i j}\times {gene}_{i}\times {gene}_{j} +{beta}_{age} \times age)$$

To determine the optimal number of G × G interactions to be included, we traversed the model constructed from 1 to 14 random G × G interactions and calculated the p-value. When satisfactory p-values can be obtained on average, the optimal model was more likely to be constructed by including the corresponding number of G × G interactions.

#### Model construction

The GG score was calculated using the optimal G × G interaction combination and incorporated in the Cox-ph model with prevalent clinicopathological features (age, gender, MRMTp methylation status) to determine independent prognostic significance. COVPRIG was constructed using age and GG score, with coefficients determined based on TCGA RNA-seq cohort for RNA-seq datasets and TCGA microarray for microarray datasets. The prognostic model was constructed and validated following the TRIPOD principle.

### Model evaluation and systemic literature review

The Cox regression analysis was performed to assess the discriminative ability of COVPRIG. IDH wild-type GBM samples were divided into three groups in ascending order of COVPRIG score, and all GBM samples were divided into four groups, with group 1 being the reference. The predictive ability of the model was evaluated using the receiver operating characteristic curve (ROC) and the corresponding AUC. Decision curves were employed to assess the net clinical benefit to patients.

To compare with other prognostic models, we searched two major databases (PubMed and Web of Science) using the following search strings: “((IDH wildtype) OR (IDHwt) OR (IDH wild-type)) AND ((glioblastoma) OR (gbm)) AND ((progn*) OR (survival)) AND ((predict*) OR (auc) OR (area under the curve) OR (receiver operator characteristic curve) OR (c-index) OR (c statistic) OR (roc) OR (calibration))”. All studies of the development and validation of IDH wild-type GBM prognostic models were included. Publications were restricted to being written in English and published before Aug 30, 2022. The exclusion criteria were set as 1) not histological GBM, 2) without a declaration of IDH wild-type, or with a mixture of IDH wild-type and mutation samples, 3) models also included WHO grade II and III gliomas, 4) models without overall survival as the outcome, 5) without evaluation of predictive efficacy, 6) conference abstracts, commentaries, editorials, or letters. Systematic review was conducted following the PRISMA principle.

### Bioinformatics

R packages ‘edgeR’ and ‘limma’ were employed to calculate the differentially expressed genes (DEGs) [37, 38]. Gene ontology (GO) and Kyoto Encyclopedia of Genes and Genomes (KEGG) enrichment analysis was performed using the online tool Metascape (https://metascape.org) [39]. The abundance of immune infiltration was deconvoluted using CIBERSORT algorithm [40]. Tumor Immune Dysfunction and Exclusion (TIDE) was employed to [35] estimate T cell function and potential sample responsiveness to immune checkpoint inhibitors (ICI) [22].

Single-cell transcriptome profiles of primary GBM were processed using the R package 'Seurat' (v4.3.0) [41, 42]. In short, genes that were expressed in less than three cells and cells that did not express over 300 genes were excluded. Expression matrices underwent independent quality control prior to integration. Batch effects were corrected by canonical correlation analysis (CCA) and mutual nearest neighbors (MNN) for the three 10 × single-cell transcriptome expression profiles [43]. Potential doublets were identified using the R package ‘DoubletFinder’ at a criteria of credible 4% [44]. Previously identified conserved tumor cell marker genes were employed to define cell identity [8]. Gene set activity was assessed using ‘AUCell’ [45]. The correlation of cell identity and COVPRIG risk-group was analyzed using ‘Scissor’ [46]. Cell–cell communication analysis was performed based on the R package ‘CellChat’ [47]. R package ‘SCENIC’ and Cytoscape software were employed for inferring and constructing the transcriptional network [45, 48].

### Statistics

All statistics were based on R software (v4.2.1). Continuous variables were summarized using mean and standard deviation, and categorized variables were described by frequency and proportion. Gene main effects and G × G interactions of prognostic significance were screened using the Cox-ph model. Kaplan–Meier (K–M) analysis and log-rank test were performed to exhibit survival differences. ROC curves and corresponding AUC, C-index, and decision curves were used to assess the predictive validity of the model. Integrated discriminative improvement was calculated for comparation of models. Wilcoxon test was employed to compare non-normally distributed continuous variables. Fisher’s exact test was performed to compare the composition ratio. A two-sided p-value <  = 0.05 was considered statistically significant when no additional declaration was made.

## Results

### Screening of G × G interactions and construction of COVPRIG

**Fig. 1:**
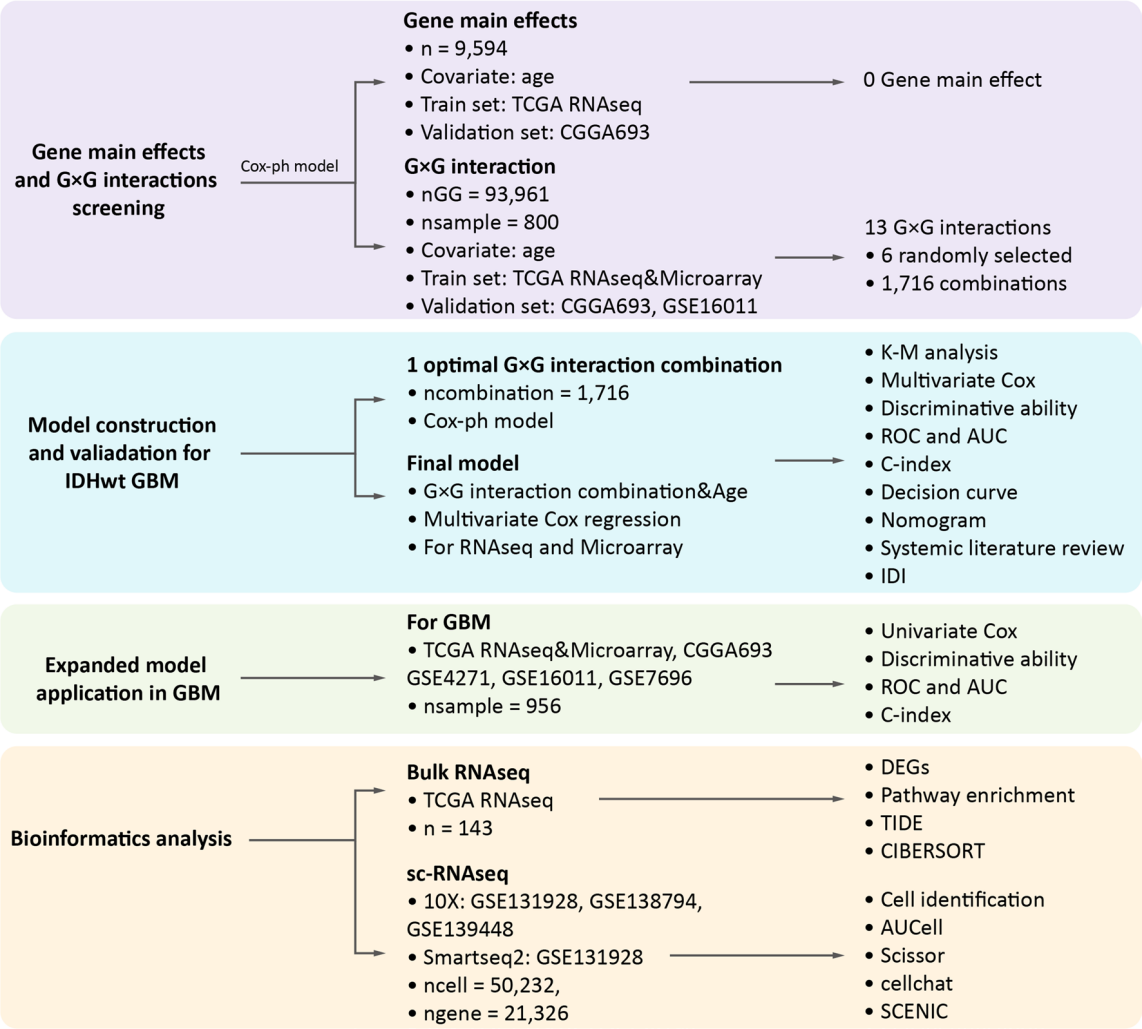
Overview of the workflow

The workflow of this study was exhibited (Fig. 1). The only gene main effect of independent prognostic significance was *KIAA1671* (multi-Cox p = 0.007), which was eliminated by p-value correction (adjust p = 0.08) (Additional file 7: Tables S3 and S4). In distilling for G × G interactions, 434 highly variable genes yielded 93,961 G × G interactions. Univariate cox regression analysis yielded 13 G × G interactions of independent prognostic significance by taking the intersection of TCGA RNAseq and microarray (Additional file 7: Table S5). Given that the prognostic performance of individual G × G interaction was not superior to that of single gene main effect, the number of G × G interactions to be included was determined. We tried combinations from a single G × G interaction to a maximum of 14 G × G interaction and assessed the performance of the model. As a result, the log-rank test p-value of the model approached 0 when over 5 G × G interactions were included simultaneously (Additional file 1: Fig. S1). Therefore, a combination of 6 G × G interactions seemed to be sufficient. 1716 combinations consisting of any 6 of the 13 G × G interactions were traversed to find the optimal one. By ranking the uniCox p-values ascendingly and taking intersections in the TCGA RNA-seq and microarray, combination No. 599 was the most statistically significant, which included *RIT2* × *OAS1*, *HOXA5* × *MLLT11*, *SLC1A2* × *FAM189A2*, *LOXL1* × *NCAPG*, *C21orf62* × *GOLGA8A*, and *UBE2S* × *NRXN1*. Based on the coefficients derived from TCGA RNA-seq and microarray respectively, these G × G interactions were assembled into the GG score using Eq. 1 (for RNA-seq) and 2 (for microarray). K–M and univariate Cox analysis disclosed that an increased GG score was suggestive of an unfavorable outcome, and ROC analysis depicted a good predictive performance (Additional file 2: Fig. S2A, B). Given the universal association between age and the prognosis of GBM (Additional file 2: Fig. S2C), the COVPRIG was constructed based on Eqs. 3 and 4 using coefficients derived from the TCGA RNA-seq and microarray, respectively.

1 $$GG Score=0.518\times RIT2+0.170 \times OAS1-0.135\times RIT2\times OAS1-0.935\times HOXA5-0.322\times MLLT11+0.178\times HOXA5\times MLLT11-0.318\times SLC1A2-0.623\times FAM189A2+0.163\times SLC1A2\times FAM189A2+0.846\times LOXL1+0.593\times NCAPG-0.253\times LOXL1\times NCAPG-0.479\times C21orf62-0.570\times GOLGA8A+0.166\times C21orf62\times GOLGA8A-0.399\times UBE2S-1.032\times NRXN1+0.254\times UBE2S\times NRXN1$$

2 $$GG Score=0.435\times RIT2+0.394 \times OAS1-0.056\times RIT2\times OAS1-0.734\times HOXA5-0.472\times MLLT11+0.074\times HOXA5\times MLLT11-0.483\times SLC1A2-0.558\times FAM189A2+0.076\times SLC1A2\times FAM189A2+0.480\times LOXL1+0.313\times NCAPG-0.066\times LOXL1\times NCAPG-0.479\times C21orf62-0.541\times GOLGA8A+0.064\times C21orf62\times GOLGA8A-0.682\times UBE2S-0.717\times NRXN1+0.104\times UBE2S\times NRXN1$$

3 $${COVPRIG}_{RNA-seq}=1.101\times GG score+0.042\times Age$$

4 $${COVPRIG}_{Microarray}= 0.535\times GG score+0.025\times Age$$

### The prognostic significance of COVPRIG

**Fig. 2:**
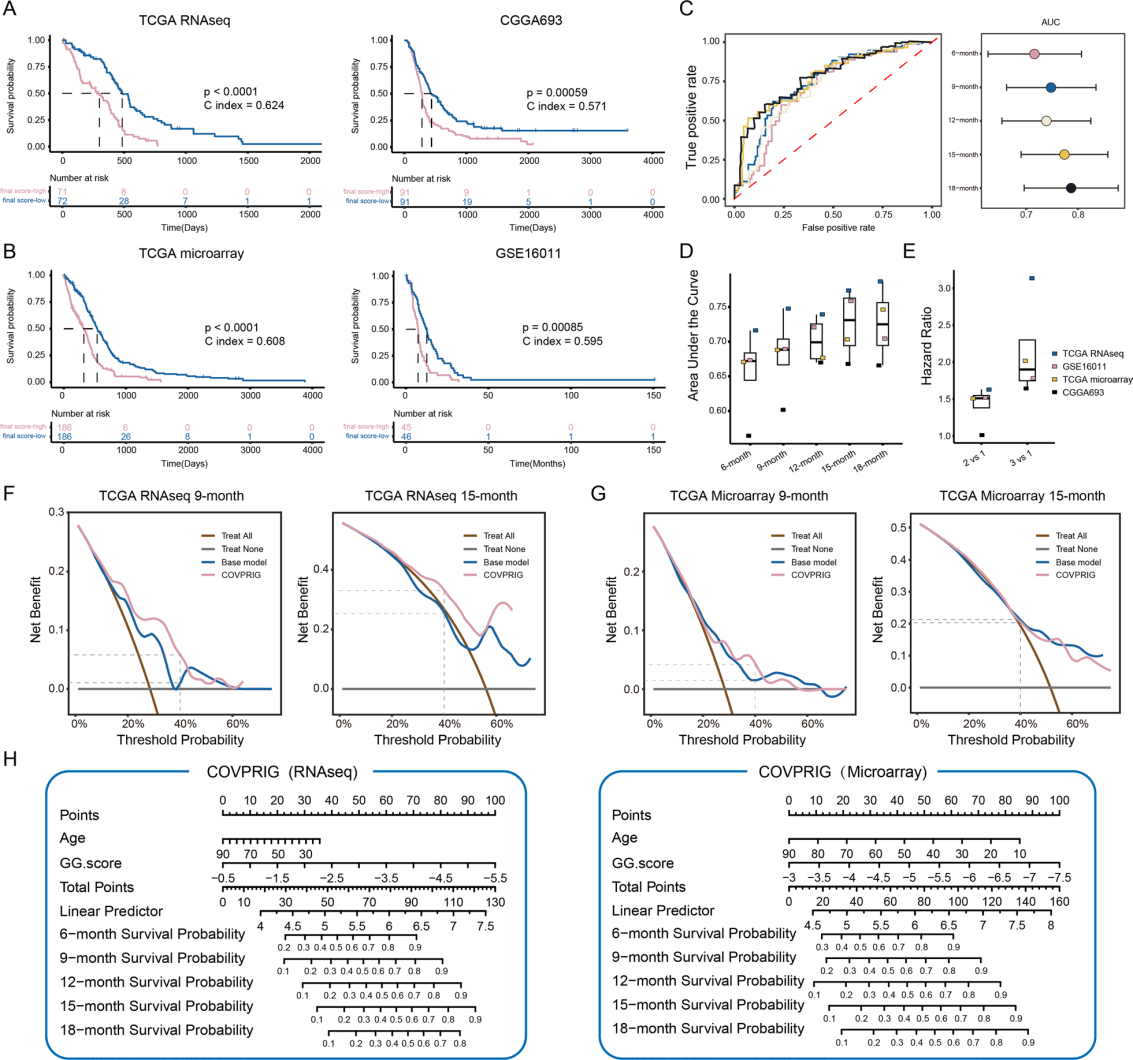
Construction and evaluation of COVPRIG. K–M analysis of the **A** TCGA RNAseq (train) and CGGA693 (external validation) and **B** TCGA microarray (train) and GSE16011 (external validation). **C** ROC curves based on the TCGA RNAseq cohort. **D** AUCs of the 4 cohorts. **E** The discriminative ability of COVPRIG. Group 1 was set as the reference. Decision curves based on the **F** TCGA RNAseq and **G** TCGA microarray cohorts. **H** The Nomogram for RNAseq and microarray-based cohorts

To demonstrate the prognostic significance of COVPRIG, samples were split into high- and low-risk groups by the median value. K–M analysis found that an increased COVPRIG score was suggestive of a significantly decreased overall survival (maximum C-index = 0.642 in the TCGA RNAseq cohort) (Fig. 2A, B). The ROC curves depicted that the prediction performance of COVPRIG was satisfactory in the TCGA RNA-seq cohort, with a minimum AUC of 6-month survival over 0.7 and a maximum of 18-month being 0.79, while humbly in CGGA693 (Fig. 2C, D). For GSE16011 and TCGA microarray, the highest AUC value exceeded 0.75 (GSE16011, 15-month survival). As for discriminative ability, samples were split into three equal groups with group 1 being the reference. The hazard ratio (HR) showed a dose–response association with groups (Fig. 2E). For example, the HR_group3vs.1_ was 3.14 in the TCGA RNA-seq cohort, higher than HR_group2vs.1_ (1.63). Decision curves depicted that COVPRIG offered more net benefit than the base model containing age, gender, IDH gene mutation, and MGMTp methylation status at 9- and 15-month survival, especially for the RNA-seq datasets (Fig. 2F, G). In sum, nomograms were constructed based on RNA-seq and microarray datasets for individualized prognostic prediction (Fig. 2H).

As IDH wild-type GBM accounts for the majority of GBM samples, the performance of COVPRIG in the entire GBM (including IDH mutant) was also tested. A total of 956 samples were collected from six cohorts. Univariate Cox analysis found that an increased COVPRIG score predicted unfavorable outcome (Additional file 2: Fig. S2D). In accordance with findings in IDH wild-type samples, the overall survival decreased significantly with increased quantile groups (Additional file 2: Fig. S2E, H). Further, ROC analysis showed satisfactory predictive performance of COVPRIG. The AUC values at 12-month or later were above 0.7 in GSE16011, TCGA microarray, and GSE7696 (Additional file 2: Fig. S2F, G). Therefore, COVPRIG was also applicable to GBM with unknown IDH mutation status.

### Comparison of COVPRIG with existing models

**Fig. 3:**
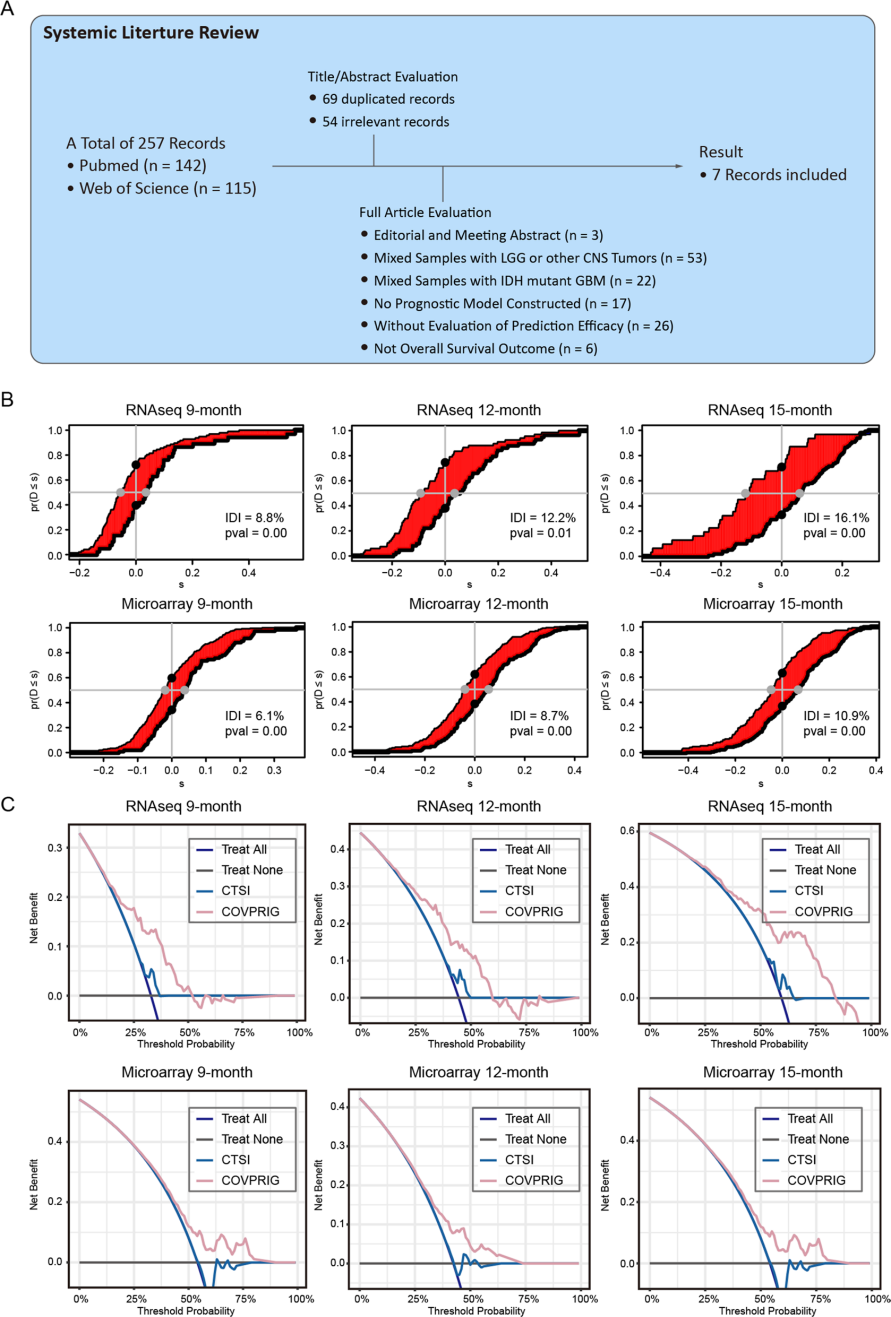
A systemic review of prognostic models of IDH wild-type GBM. **A** Screening of previous prognostic models. **B** Improvement of COVPRIG in contrast to another mRNA-based externally validated model, CTSI. **C** Decision curves of COVPRIG and CTSI

To compare with previous studies, a systemic literature review was conducted. A total of 258 records were collected from the Pubmed and Web of Science. 69 duplicate and 54 irrelevant records were excluded by evaluating titles and abstracts. Through full-text assessment, editorial and conference abstracts (n = 3), a mixture with LGG or other central nervous system (CNS) tumors (n = 53), a mixture with IDH mutant GBM (n = 22), no prognostic model constructed (n = 17), without evaluation of the predictive ability (n = 26), and not overall survival as the outcome variable (n = 6) were excluded, resulting in 7 eligible studies [14–20] (Fig. 3A). Three studies developed prognostic models using transcriptome profiles, other models were developed based on either multi-omics data, MGMTp status, laboratory, or imaging parameters. All models were constructed primarily using the Cox-ph model, with two additionally employed LASSO, and one SVM. Five models employed datasets retrieved from the TCGA or CGGA databases, including COVPRIG. The largest train set included 404 samples, but was only internally validated. 2 of the models were not validated by either internal or external datasets. None of the models included more than 1000 samples overall.

The basic information, AUC and C-index of these models were extracted and curated in Table 1. Generally, models performed better in the train set. The multi-Cox HR (high-risk vs. low-risk) for the seven models ranged from 2.11 to approximately 50. Model 5 reported an extremely high HR of around 50 in the train set. Models 3, 6, and 8 did not report independent prognostic significance. The predictive accuracy varied, with AUCs ranging from 0.58 to 0.87. Models 4 and 6 reported higher AUCs, with 12-month AUCs being 0.78 and 0.74. The 12-month AUCs of COVPRIG ranged from 0.67 to 0.74, on par with model 6. C-indexes were calculated for models 3 and 8 only, both were higher than the COVPRIG. Model 4 (CTSI) performed well and robust among the externally validated models, and, when calibration to the same condition, a 6–16% improvement in COVPRIG in predicting 9–15-month survival was found (Fig. 3B, C), indicating that COVPRIG outperformed CTSI.

**Table 1:** Systemic review of the prognostic model for IDH wildtype GBM

No	PMID	Method	Description	Ethnicity	Validation type	Data source		Sample size			Performance in train set		Performance in validation set	
						Train	Validation	Train	Validation	Total	AUC	C-index	AUC	C-index
1		Cox	mRNA and age-based model	Tans-ethnic	External	TCGA_RNAseq_, TCGA_microarray_	CGGA693 GSE16011	143 + 372	190 + 95	800	AUC_18-month_ = 0.79	0.62	AUC_18-month_ = 0.70	0.60
2	34830935	Cox	neutrophil-to-lymphocyte ratio			Local		85		85	AUC_NLR_ = 0.58AUC_dNLR_ = 0.62			
3	35610333	SVM, Cox	Multi-omics data		Internal	Local	Local	404	112	516	0.75	0.75	0.63	
4	31632497	Cox	mRNA-based model	Tans-ethnic	Internal, External	TCGA_RNA-seq_	TCGA_microarray_, GEO, CGGA, Local	195	529, 54, 144, 178	905	0.82			
5	31921684	Cox, LASSO	mRNA-based model	Tans-ethnic	External	CGGA	TCGA_RNAseq_ TCGA_microarry_	105	139 386	630	AUC_12-month_ = 0.78		AUC_12-month_ = 0.74	
6	32851059	Cox	Lnc-RNA-based model	Tans-ethnic	External	TCGA CGGA325	CGGA693				AUC_12-month_ = 0.74 AUC_36-month_ = 0.87		AUC_12-month_ = 0.55 AUC_36-month_ = 0.73	
7	31167648	Cox	MGMT promoter methylation			Local		111		111	0.77			
8	35153659	LASSO Cox	MRI and clinical features	Tans-ethnic	Internal	TCGA Local		100	42	142		0.84		0.80

### Molecular underpinnings of COVPRIG

We further interrogated the molecular underpinnings associated with the COVPRIG-based risk groups. Functional enrichment analysis based on DEGs disclosed that genes up-regulated in the high-risk group were enriched in signaling pathways associated with ECM, and genes up-regulated in the low-risk group were primarily enriched in the interferon alpha response pathway (Additional file 3: Fig. S3A, B). IDH wild-type GBM has been divided into four transcriptome-based subtypes [49]. The low-risk group tended to have more classical and less proneural samples (Additional file 3: Fig. S3C). Instead, COVPRIG identified samples exhibiting a more unfavorable prognosis in ME, CL, and NE subtypes (Additional file 3: Fig. S3D). Deconvolutional algorithm identified higher proportion of regulatory T cells and M0 macrophages in the high-risk group, indicating an immunosuppressive TME (Additional file 3: Fig. S3). Several immune-related gene signatures have been curated (Additional file 7: Table S6), and ROC curves disclosed that the COVPRIG score gave the best performance in predicting the regulatory T cell (Additional file 3: Fig. S3F), in line with the findings of CIBERSORT. Moreover, the TIDE algorithm identified significantly more samples with potential responsiveness to the ICI therapy in the low-risk group (p-value = 0.046) (Additional file 3: Fig. S3G). Collectively, these results indicated that the TME of the COVPRIG high-risk group was more immunosuppressive.

**Fig. 4:**
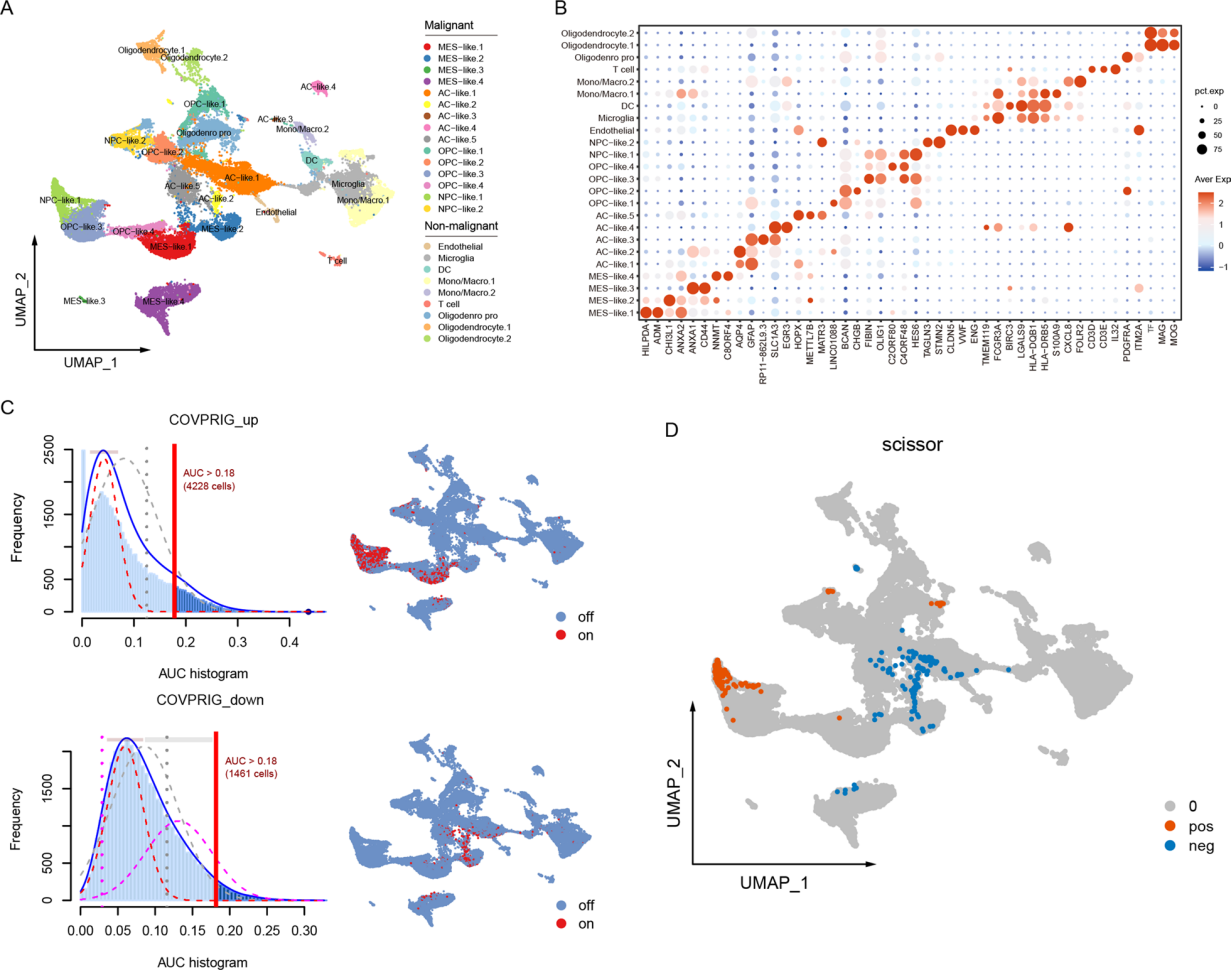
Association between NPC-like tumor cell with poor outcome. **A** Cell types and **B** marker genes of each type of cell. **C** The activity COVPRIG_up (DEGs with log2FC > 0.35 and p.val < 0.05, ngene = 73) and COVPRIG_down (log2FC > 0.35, p.val < 0.05, ngene = 94) in each type of cell. **D** Scissor algorithm assessed the correlation between COVPRIG-based risk groups and specific cell types. pos: positively correlated, neg: negatively correlated

To gain further insight, three scRNA-seq datasets of primary GBM were integrated for downstream analysis (Additional file 4: Fig. S4A, B). Potential doublets were excluded, resulting in a total of 50,232 quantified cells (Additional file 4: Fig. S4C). After cell circle normalization, 24 clusters were initially identified (Fig. 4A, B, Additional file 4: Fig. S4D) and were defined as 4 types of malignant cells (neural-progenitor-like/NPC, oligodendrocyte-progenitor-like/OPC, astrocyte-like/AC, and mesenchymal-like/MES), endothelial, microglial, mono/macro, T cell and oligodendrocyte, according to previously well-defined marker genes [8]. As enrichment analysis suggested an alteration of ECM-associated pathways in the COVPRIG high-risk group, the activity of ECM-related pathways of each type of cell was estimated using AUCell. As a result, ECM-related pathways were associated with mono/macro (NABA matrisome-associated, NABA ECM affiliated), and to a less extent, with MES-like cells (NABA core matrisome, Reactome extracellular matrix organization) (Additional file 5: Fig. S5), indicating a role of these cells in shaping the ECM. Besides, the top up- and down-regulated genes were also curated as the signatures of the COVPRIG high- and low-risk groups, which were mainly enriched in NPC-like.1/OPC-like.3/MES-like.1 and MES-like.2/AC-like.1/2/5 cells, respectively (Fig. 4H, I). These findings can be further validated by an independent Smartseq2-based single-cell RNAseq dataset (Additional file 6: Fig. S6A–C). Interestingly, Scissor algorithm found that NPC-like.1 and to a lesser extent, OPC-like.3 were most correlated with the COVPRIG high-risk group, while the opposite was true for a subset of MES-like.2 and AC-like.1/2/5 cells (Fig. 4D), in accordance with the AUCell. Together, these results highlighting the role of NPC-like.1 tumor cells in IDH-wild type GBM.

**Fig. 5:**
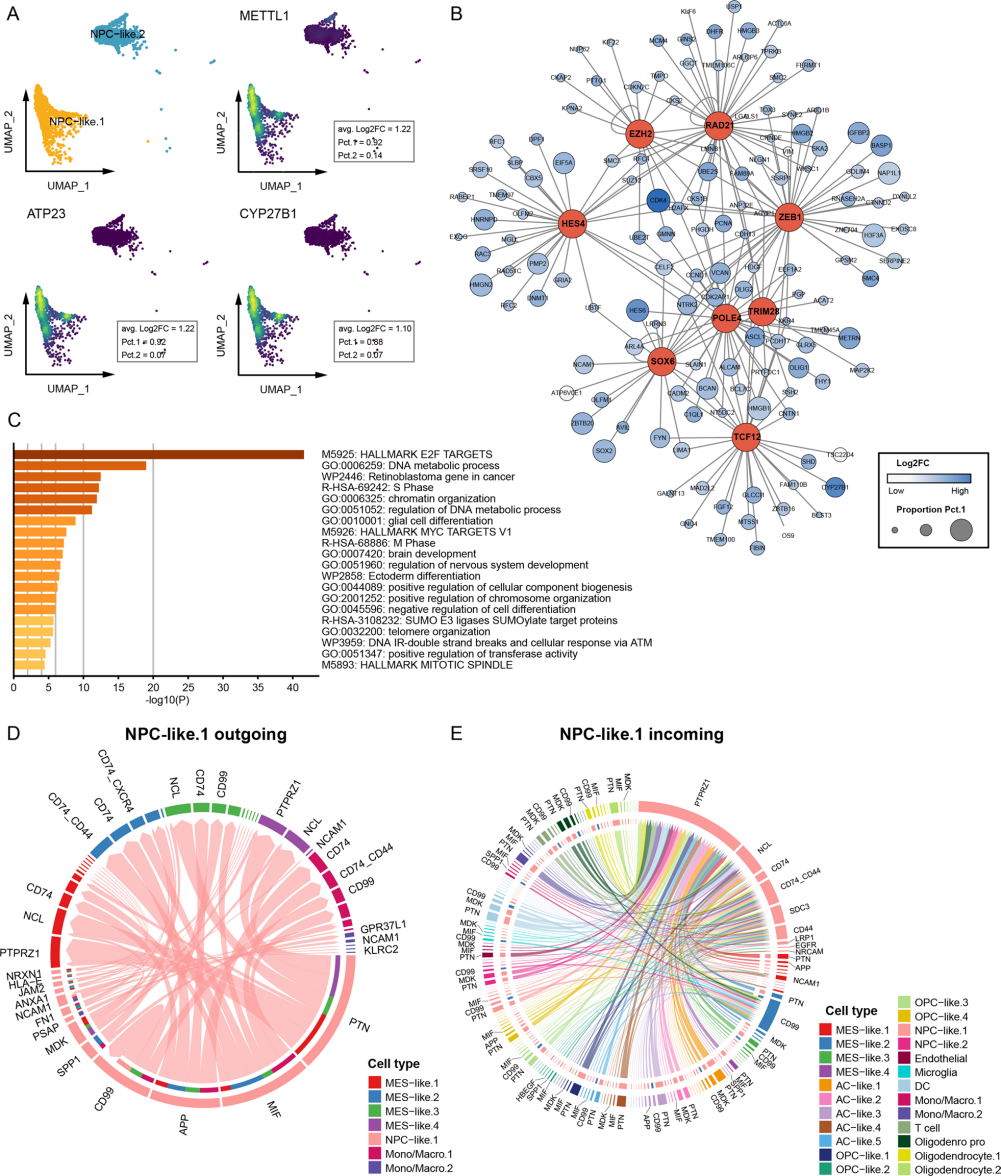
Features of NPC-like.1. **A** Genes uniquely expressed by NPC-like.1 cells and their expression density. **B** Transcriptional regulatory network and their high confidence target genes of NPC-like.1. The size of the node is proportional to the percentage of cells expressing the gene and the color is proportional to the average expression. **C** Enrichment analysis of high confidence target genes. **D** Signal export from NPC-like.1 cell to MES-like cells and monocytes/macrophages and important molecules. **E** Signal input to NPC-like.1 cell from other cells and important molecules

Two subsets of NPC-like tumor cells were identified. NPC-like.1 uniquely expressed METTL1, ATP23, and CYP27B1, and had different transcriptional regulation network from NPC-like.2 (Fig. 5A, B, Additional file 6: Fig. S6D, E). High-confidence target genes of the NPC-like.1 transcriptional factors were primarily enriched in the E2F targets (Fig. 5C), which is associated the cell cycle controlling. NPC-like cells are implicated in tumor invasion [26], and in this scenario, it was mainly monocytes/macrophages and some MES-like cells that involved in the modification of ECM. Therefore, NPC-like.1 may be involved in ECM modification by regulating the function of these cells. Cellchat found that NPC-like.1 interact with MES-like cells and monocytes/macrophages mainly in a PTN, MIF, APP and CD99-dependent manner (Fig. 5D, Additional file 6: Fig. S6F), with MIF and APP playing a role in ECM remodelling [50, 51]. On the other hand, PTPRZ1 played an essential role in receiving signaling from outside and therefore may be a potential target for regulating the function of NPC-like.1.

## Discussion

Multiple mechanisms dominate the heterogeneous prognosis of IDH wild-type GBM patients. Due to the lack of extremely effective therapy for GBM, it is worthwhile for clinicians to estimate the mortality risk and decide on available therapies to maximize the clinical benefit for individuals. Obstacles include the limited data available for study, and potential geographic and demographic disparities [52]. Herein, we constructed a prognostic model using G × G interactions that greatly increased the number of common candidates of prognostic significance between small sample datasets. The model was made robust through a multi-step screening process and separate determination of coefficients from RNA-seq and microarray data, as confirmed by systematic performance comparison. Collectively, this study provided a reference for accurately determining the prognosis of IDH wild-type GBM.

In total, six gene pairs were used to construct COVPRIG, including *RIT2* and *OAS1*, *HOXA5* and *MLLT11*, *SLC1A2* and *FAM189A2*, *LOXL1* and *NCAPG*, *C21orf62* and *GOLGA8A*, and *UBE2S* and *NRXN1*. The biological implications of some of these genes in GBM have been explored. *OAS1* is an interferon-inducible gene that encodes a protein involved in the synthesis of 2',5'-oligoadenylates and the innate immune response. *OAS1* may affect the prognosis of GBM in a *TRIM5*-dependent manner [53]. Increased expression of *HOXA5*, encoding a transcription factor named homeobox genes, was associated with the tumorigenic potential of glioma stem cell [54, 55]. In addition, ubiquitin-conjugating enzyme E2S encoded by *UBE2S* is associated with the activity of PI3K-Akt pathway in GBM and may serve as a therapeutic target [56]. This suggests that the candidate genes derived from our screening for G × G interactions may have biological significance in IDH wild-type GBM.

The essential role of G × G interactions in prognosis was inspired by Zhang et al. [21]. They found that the expression of *EGLN2* was negatively correlated with *HIF1A* in non-small cell lung cancer. Interestingly, *HIF1A* shifted from an independent risk factor to a protective factor as *EGLN2* expression increased. Likewise, we found that the prognostic significance of *LOXL1* and *C21orf62* was associated with the expression of *NCAPG* and *GOLGA8A* (data not shown). The mathematical representation of G × G interactions include the z-score in the Cox-ph model, and the product of normalized gene expressions [22, 23]. Zhang et al. and Chen et al. constructed effective prognostic models using the product of gene expressions as a metric for G × G interactions [23, 24]. However, these studies included 613 and 505 patients as train sets, and the total sample size was around 1000, which provided a guarantee for developing robust models. Currently, individual GBM cohorts rarely reach such a magnitude, in which poses challenges in identifying genes with shared prognostic value. Notably, G × G interaction greatly increased the number of candidates for model construction, thus, to some extent compensating for screening candidates available for more cohorts.

The performance of COVPRIG versus previously constructed models was evaluated. Some models seemed to outperform COVPRIG in terms of AUC and the C-index. However, it is difficult to avoid statistical overfitting without external validation, therefore limiting the application of some these models. In the models externally validated, COVPRIG had a 12-month AUC of 0.74, which was comparable with models 5 and 6. The highest AUC of COVPRIG occurred at 18-month (0.79), second to models 4 and 6, but still satisfactory. Notably, COVPRIG showed a 6–16% improvement relative to the externally validated and best performing model 4 after calibration to the same conditions, indicating that it should be the optimal prognostic model available.

The molecular underpinnings of COVPRIG were addressed. The dysregulated genes were mainly enriched in ECM-related signaling pathways, suggesting that COVPRIG high-risk group was characterized by ECM remodeling. Further, AUCell identified an association between ECM-associated pathways with monocytes/macrophages and some MES-like cells, in line with a previous study [57]. Interestingly, both AUCell and Scissor identified that genes up-regulated in the COVPRIG high-risk group were primarily enriched in NPC-like.1, indicating that the unfavorable outcome of the COVPRIG high-risk group was driven by NPC-like.1, and the tumor invasion associated with NPC-like cells was dependent on its interaction with cells including macrophages and MES-like cells. Further, we addressed the gene expression, transcriptional regulation, and essential cell communication molecules of NPC-like.1, which may provide a preliminary basis for subsequent studies and therapies targeting these cells.

There were several limitations. We should acknowledge that COVPRIG were not internally validated, which may lead to an under-fitting of the model. In the initial stages of exploration, tenfold cross-validation was performed instead of dividing samples into train and validation sets. Despite being able to achieve an AUC approaching 0.9 and having a satisfactory C-index in one training cohort, the excellent performance never passing on to others. This may be related to the intrinsic differences in different GBM cohorts and exploring such differences is one of the directions for future update of COVPRIG. In addition, as COVPRIG was constructed primarily with Caucasians and Asians, it should be cautiously applicated to other ethnic populations.

## Conclusions

This study provided a current robust prognostic tool for GBM which was applicable to both microarray and RNA sequencing data. In addition, this study highlighted the role of a class of neuronal progenitor cells in driving poor prognosis in GBM. These results may provide basis for future research.

### Supplementary Information

**Additional file 1: Figure S1.** The log-rank p value of the model when incorporating increasing numbers of G × G interactions. The bar plots showed an explicit trend that the prognostic efficacy of the model became more statistical significant as the number of G × G interactions incorporated into the model increased. P-values were calculated based on TCGA RNAseq. Red dashed line indicated p = 0.05.

**Additional file 2: Figure S2.** The prognostic significance of the GG score. (**A**) The K–M analysis. (**B**) AUCs of each cohort at specific time points. (**C**) Multivariate Cox regression analysis of GG score with clinicopathological features. (**D**) Univariate Cox regression of COVPRIG score in multiple GBM datasets. (**E**) Discriminative ability of COVPRIG based on TCGA microarray COVPRIG score increased from group.1 to group.4. (**F**) ROC curves based on TCGA microarray. (**G**) Predictive ability of COVPRIG. (**H**) Increased COVPRIG scores impair overall survival in a dose–response manner.

**Additional file 3: Figure S3.** Transcriptome features of COVPRIG-based group. (**A**) Volcano plot of DEGs and (**B**) functional enrichment analysis. (**C**) Distribution of transcriptome-based GBM subtypes. (**D**) K–M analysis of COVPRIG-high and -low group in different GBM subtypes. (E) Differentially infiltrated immune cells estimated by CIBERSORT. The TME of COVPRIG high-risk group contained more regulatory T cell and M0 macrophages and CD4 T cell, monocyte, and eosinophil instead. (**F**) Performance of COVPRIG score in predicting previously well studied immune-related gene signatures. (**G**) Distribution of samples that had potential to respond to ICI.

**Additional file 4: Figure S4.** Preprocess of single-cell datasets. (**A**, **B**) Before and after batch effects correction. (**C**) Identifying potential doublets using DoubletFinder. (**D**, **E**) Before and after cell circle normalization. GSE131 represents GSE131928, GSE138 represents GSE138794, GSE139 represents GSE139448.

**Additional file 5: Figure S5.** Cells associated with interested pathways. Gene signatures that were enriched in the COVPRIG high- and low-risk groups were retrieved from MSigDB. Activation status of these pathways were estimated by AUCell and automatically binarized.

**Additional file 6: Figure S6.** (**A**) Cell type of SmartSeq2-based primary GBM. AUCell identified cells that had enriched (**B**) COVPRIG_up and (**C**) COVPRIG_down gene signatures. (**D**) Transcriptional factors and regulons activated in NPC-like.1 and NPC-like.2 cells based on SCENIC. Algorithm. (**E**) Transcriptional factors activated in NPC-like.2 with their high confidence target genes. (**F**) Cellular communication-dependent molecules.

**Additional file 7: Table S1.** Demographics of IDH wild-type GBM samples included in the study. **Table S2.** Demographics of all GBM samples included. **Table S3.** The first screen for gene main effects were of prognostic significance. Genes were included in the Cox-ph model on a case-by-case basis, with age as the covariate. **Table S4.** The second screen for gene main effects based on the TCGA cohort. Genes with p values less than 0.05 in the first screen were included in the cox-ph model simultaneously, with age as a covariate. **Table S5.** 13 G × G interactions in the first screen. **Table S6.** Gene signature associated with T cell function.

## Data Availability

Data included in this study was available from the corresponding author upon reasonable request.

## Abbreviations

AC: Astrocyte-like cell
AUC: Area under the curve
CCA: Canonical correlation analysis
CNS: Central nervous system
DEG: Differentially expressed gene
ECM: Extracellular matrix
FPKM: Fragments per kilobase of exon model per million mapped fragments
GBM: Glioblastoma
HR: Hazard ratio
ICI: Immune checkpoint inhibitor
IDI: Integrated discrimination improvement
IDH: Isocitrate dehydrogenase
LGG: Lower-grade glioma
MAD: Median absolute deviation
MES: Mesenchymal-like cell
MGMT: O-6-methylguanine-DNA methyltransferase
MNN: Mutual nearest neighbors
NPC: Neural-progenitor-like cell
OPC: Oligodendrocyte-progenitor-like cell
ROC: Receiver operating characteristic curve
TERT: Telomerase reverse transcriptase
TIDE: Tumor Immune Dysfunction and Exclusion
TME: Tumor microenvironment
WHO: World health organization
