# Supplementary figures and images for: COVPRIG robustly predicts the overall survival of IDH wild-type glioblastoma and highlights METTL1+ neural-progenitor-like tumor cell in driving unfavorable outcome
Source: J Transl Med. 2023 Aug 8;21:533. doi: 10.1186/s12967-023-04382-2 (PMC10408096; doi:10.1186/s12967-023-04382-2)

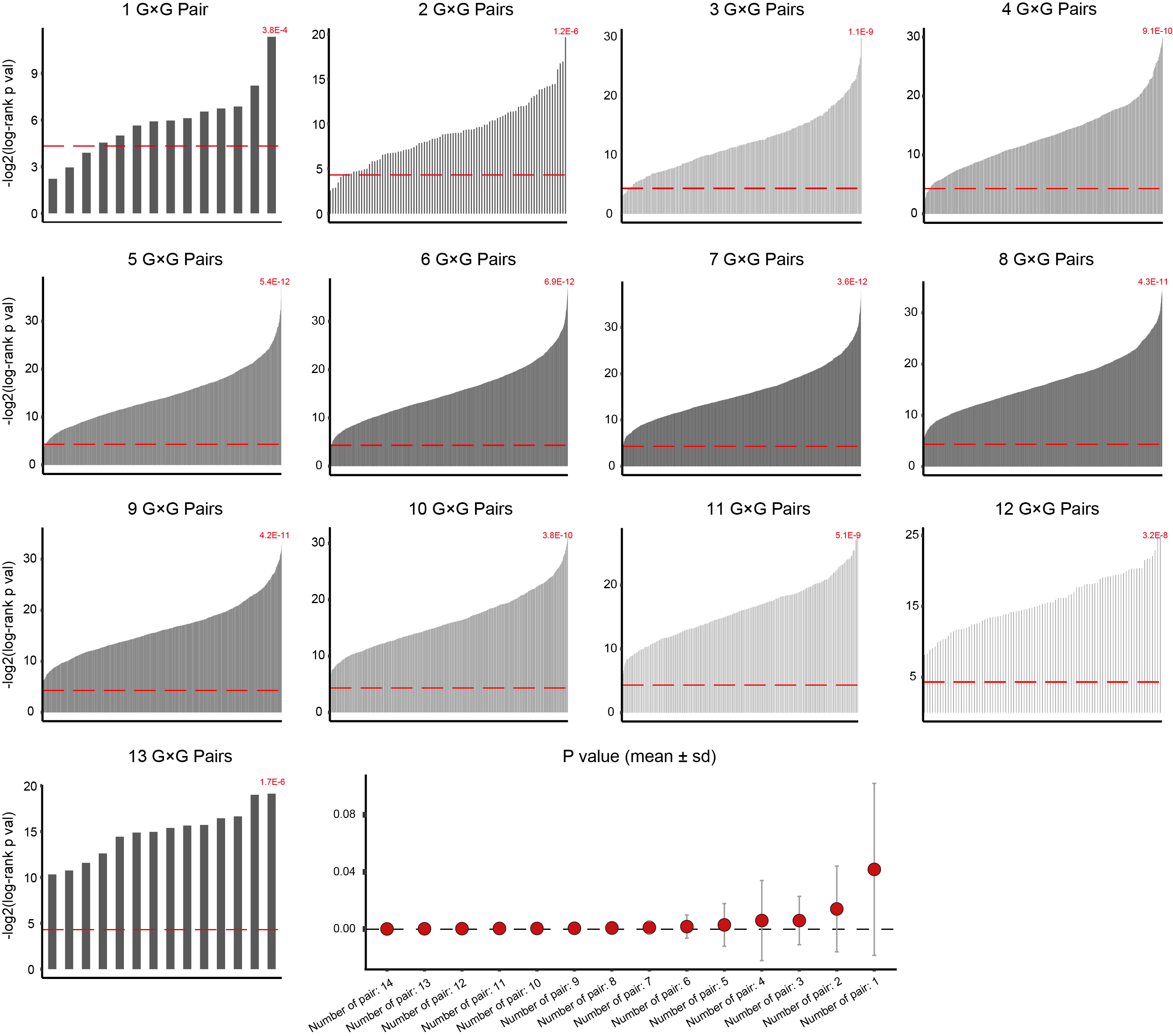

Supplement: Supplementary file 1 — Additional file 1: Figure S1. The log-rank p value of the model when incorporating increasing numbers of G × G interactions. The bar plots showed an explicit trend that the prognostic efficacy of the model became more statistical significant as the number of G × G interactions incorporated into the model increased. P-values were calculated based on TCGA RNAseq. Red dashed line indicated p = 0.05. [file 12967_2023_4382_MOESM1_ESM.tif]

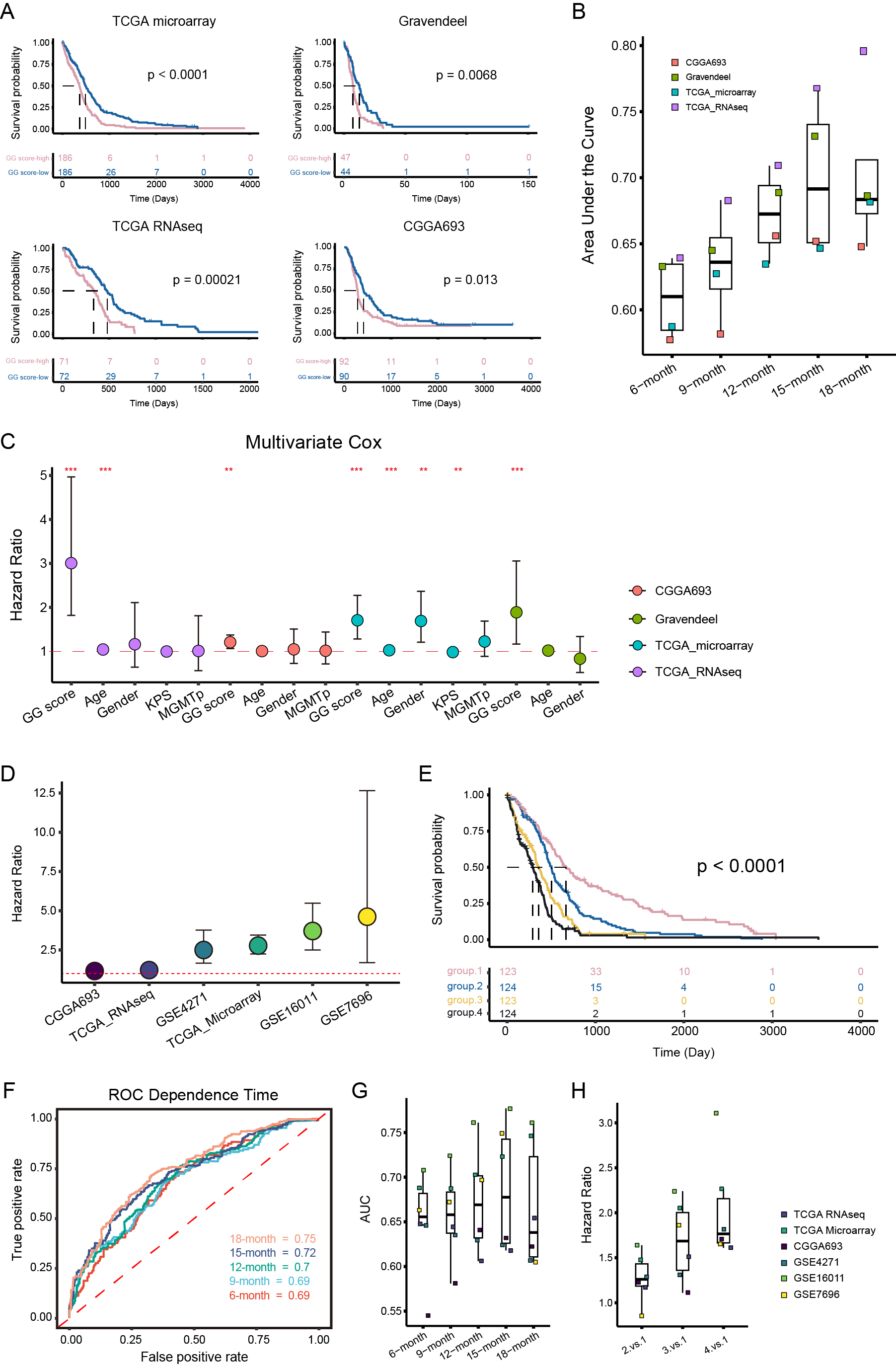

Supplement: Supplementary file 2 — Additional file 2: Figure S2. The prognostic significance of the GG score. (A) The K–M analysis. (B) AUCs of each cohort at specific time points. (C) Multivariate Cox regression analysis of GG score with clinicopathological features. (D) Univariate Cox regression of COVPRIG score in multiple GBM datasets. (E) Discriminative ability of COVPRIG based on TCGA microarray COVPRIG score increased from group.1 to group.4. (F) ROC curves based on TCGA microarray. (G) Predictive ability of COVPRIG. (H) Increased COVPRIG scores impair overall survival in a dose–response manner. [file 12967_2023_4382_MOESM2_ESM.tif]

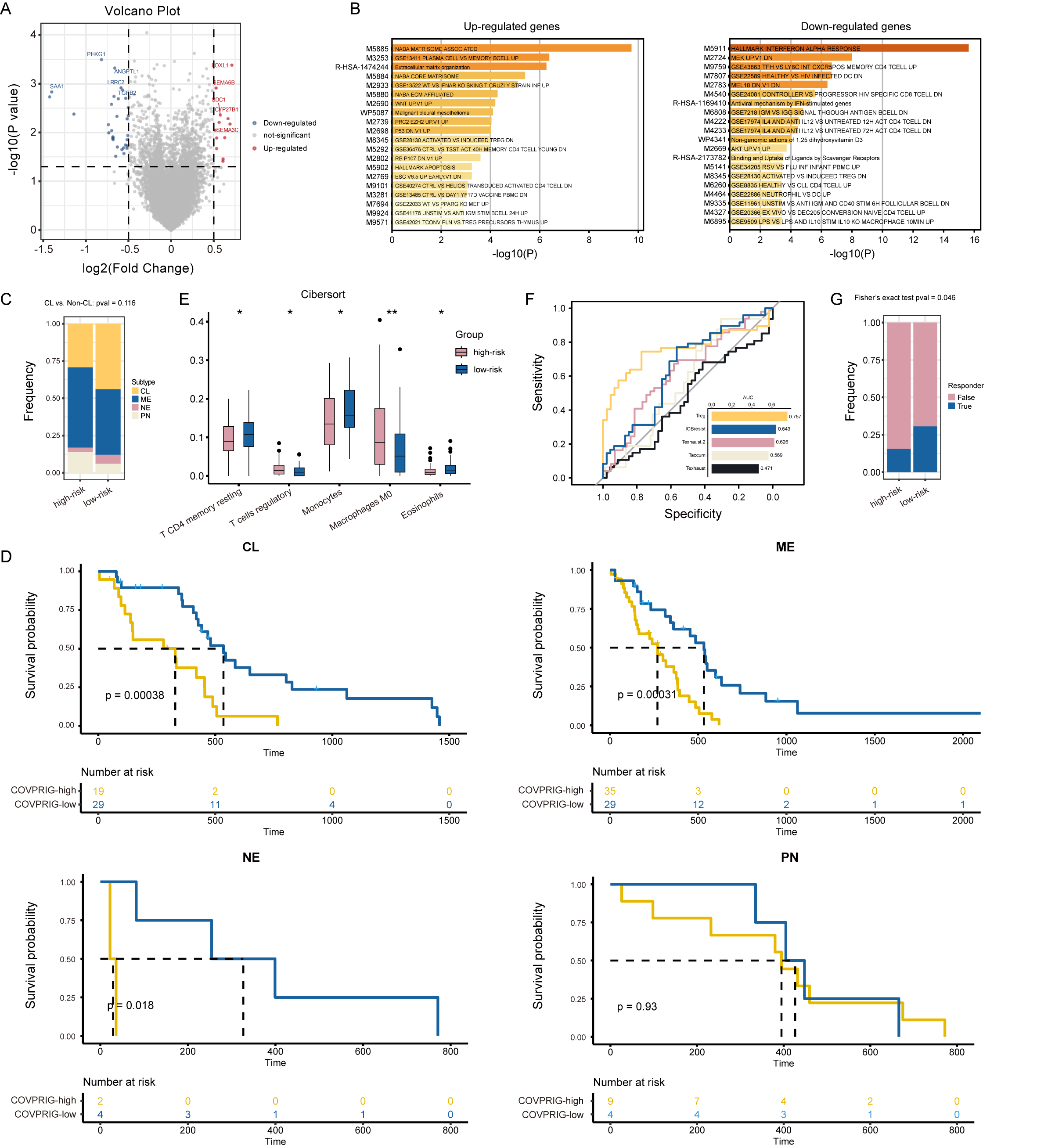

Supplement: Supplementary file 3 — Additional file 3: Figure S3. Transcriptome features of COVPRIG-based group. (A) Volcano plot of DEGs and (B) functional enrichment analysis. (C) Distribution of transcriptome-based GBM subtypes. (D) K–M analysis of COVPRIG-high and -low group in different GBM subtypes. (E) Differentially infiltrated immune cells estimated by CIBERSORT. The TME of COVPRIG high-risk group contained more regulatory T cell and M0 macrophages and CD4 T cell, monocyte, and eosinophil instead. (F) Performance of COVPRIG score in predicting previously well studied immune-related gene signatures. (G) Distribution of samples that had potential to respond to ICI. [file 12967_2023_4382_MOESM3_ESM.tif]

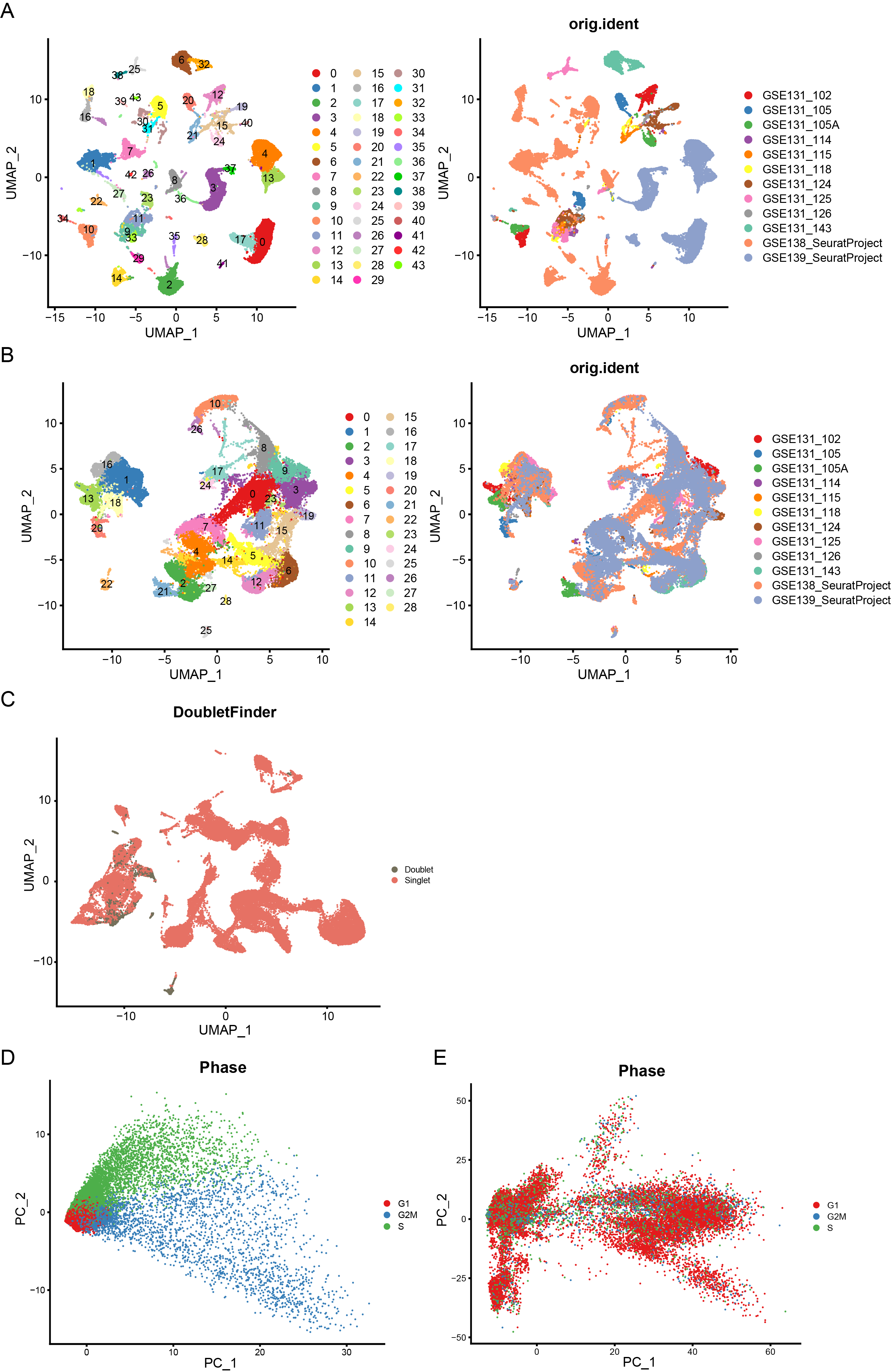

Supplement: Supplementary file 4 — Additional file 4: Figure S4. Preprocess of single-cell datasets. (A, B) Before and after batch effects correction. (C) Identifying potential doublets using DoubletFinder. (D, E) Before and after cell circle normalization. GSE131 represents GSE131928, GSE138 represents GSE138794, GSE139 represents GSE139448. [file 12967_2023_4382_MOESM4_ESM.tif]

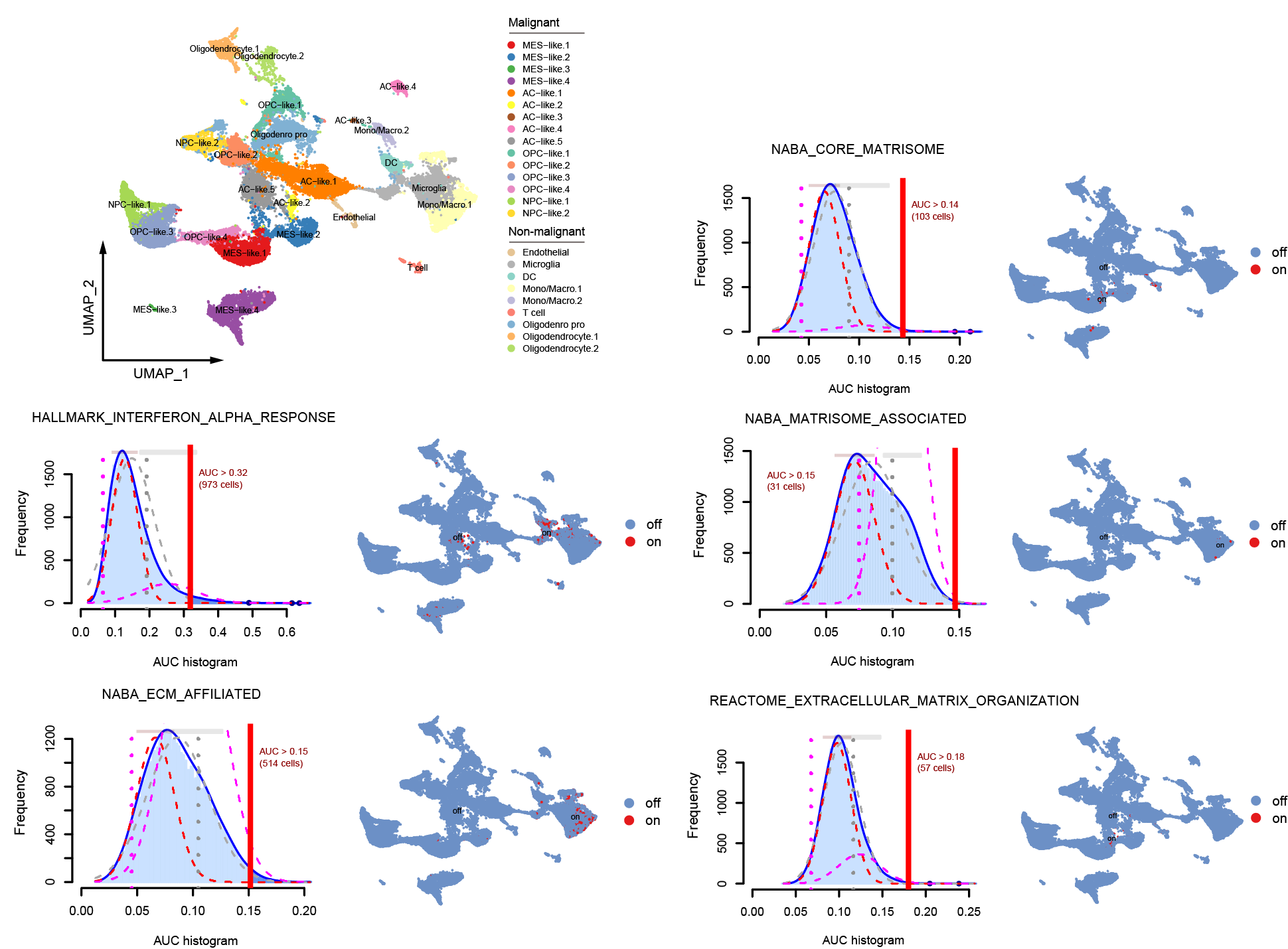

Supplement: Supplementary file 5 — Additional file 5: Figure S5. Cells associated with interested pathways. Gene signatures that were enriched in the COVPRIG high- and low-risk groups were retrieved from MSigDB. Activation status of these pathways were estimated by AUCell and automatically binarized. [file 12967_2023_4382_MOESM5_ESM.tif]

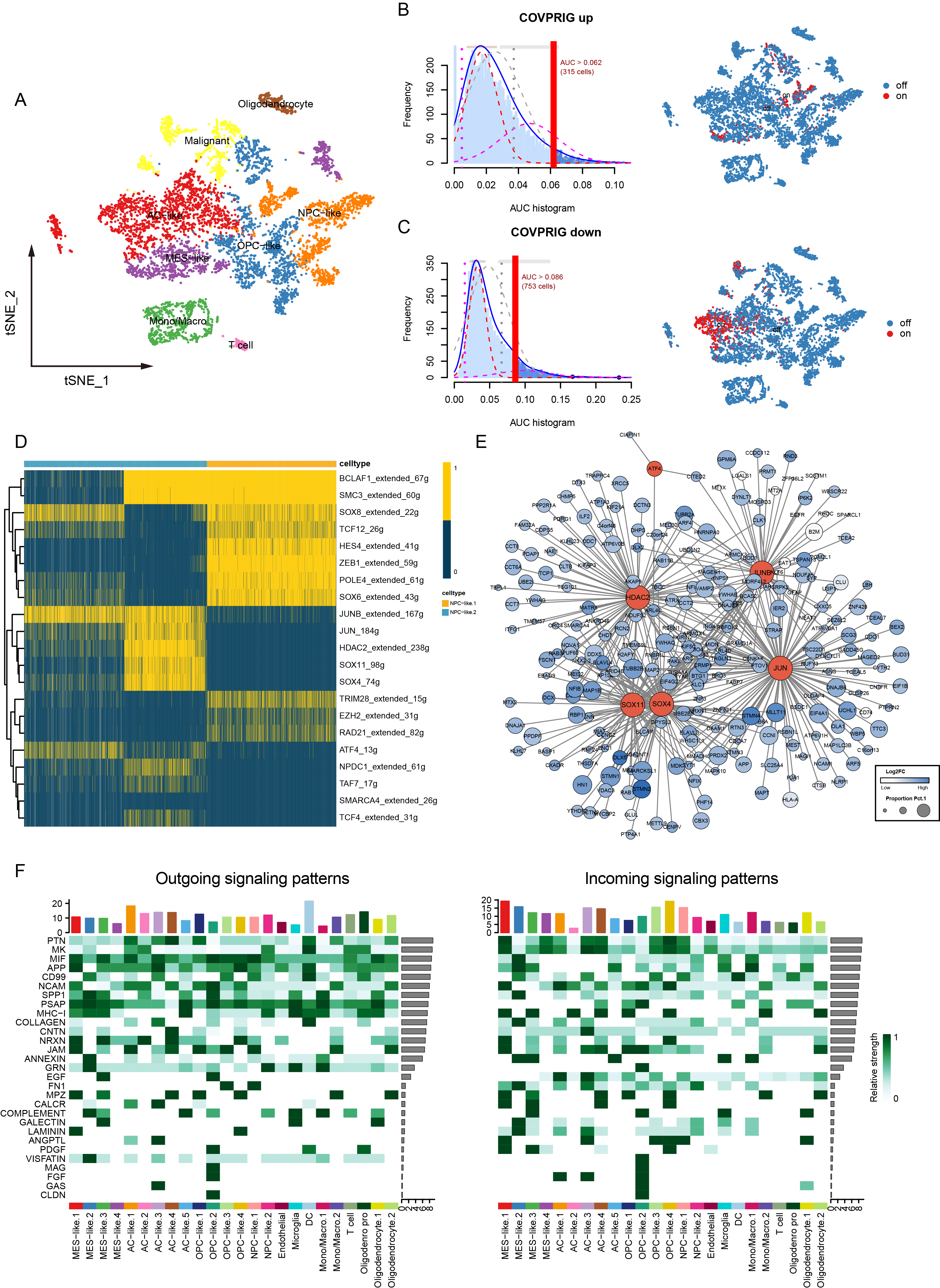

Supplement: Supplementary file 6 — Additional file 6: Figure S6. (A) Cell type of SmartSeq2-based primary GBM. AUCell identified cells that had enriched (B) COVPRIG_up and (C) COVPRIG_down gene signatures. (D) Transcriptional factors and regulons activated in NPC-like.1 and NPC-like.2 cells based on SCENIC. Algorithm. (E) Transcriptional factors activated in NPC-like.2 with their high confidence target genes. (F) Cellular communication-dependent molecules. [file 12967_2023_4382_MOESM6_ESM.tif]
