# Supplementary material for: COVPRIG robustly predicts the overall survival of IDH wild-type glioblastoma and highlights METTL1+ neural-progenitor-like tumor cell in driving unfavorable outcome
Source: J Transl Med. 2023 Aug 8;21:533. doi: 10.1186/s12967-023-04382-2 (PMC10408096; doi:10.1186/s12967-023-04382-2)
Supplement: Supplementary file 7 — Additional file 7: Table S1. Demographics of IDH wild-type GBM samples included in the study. Table S2. Demographics of all GBM samples included. Table S3. The first screen for gene main effects were of prognostic significance. Genes were included in the Cox-ph model on a case-by-case basis, with age as the covariate. Table S4. The second screen for gene main effects based on the TCGA cohort. Genes with p values less than 0.05 in the first screen were included in the cox-ph model simultaneously, with age as a covariate. Table S5. 13 G × G interactions in the first screen. Table S6. Gene signature associated with T cell function. [file 12967_2023_4382_MOESM7_ESM.docx]

Table S1. Demographics of IDH wild-type GBM samples included in the study.

| **Characteristics** | **TCGA RNAseq** | **TCGA Microarray** | **CGGA693** | **GSE16011** | **Overall** |
| --- | --- | --- | --- | --- | --- |
|  | **Train** | **Train** | **Testing** | **Testing** |  |
| **Data type** | RNAseq | Microarray | RNAseq | Microarray |  |
| **Number of samples** | 143 | 372 | 190 | 95 | 800 |
| **Age at diagnosis (years)** | 61.67±11.88 | 60.12±12.84 | 50.56±14.25 | 55.47±13.37 | 56.96±14.09 |
| **Gender (%)** |  |  |  |  |  |
| Male | 92 (64.34) | 229 (61.56) | 111 (58.42) | 68 (71.58) | 500 (62.5) |
| Female | 51 (35.66) | 143 (38.44) | 79 (41.58) | 27 (28.42) | 300 (37.5) |
| **1p/19q (%)** |  |  |  |  |  |
| Codeleted | 0 (0) | 0 (0) | 4 (2.11) | 5 (5.26) | 9 (1.13) |
| Non-codeleted | 139 (97.20) | 362 (97.31) | 160 (84.21) | 35 (36.84) | 696 (87) |
| Unknown | 4 (2.80) | 10 (2.69) | 26 (13.68) | 55 (57.89) | 95 (11.88) |
| **MGMT promoter (%)** |  |  |  |  |  |
| Methylated | 43 (30.07) | 112 (30.11) | 83 (43.68) | - | 238 (29.75) |
| Un-methylated | 69 (48.25) | 164 (44.09) | 72 (37.89) | - | 305 (38.13) |
| Unknown | 31 (21.68) | 96 (25.81) | 35 (18.42) | - | 162 (20.25) |
| **Radiotherapy (%)** |  |  |  |  |  |
| Yes | 16 (11.19) | 298 (80.11) | 149 (78.42) | - | 463 (57.88) |
| No | 66 (46.15) | 55 (14.78) | 26 (13.68) | - | 147 (18.38) |
| Unknown | 61 (42.66) | 19 (5.11) | 15 (7.89) | - | 95 (11.88) |
| **Pharmacotherapy (%)** |  |  |  |  |  |
| Yes | 52 (36.36) | 134 (36.02) | 154 (81.05) | - | 340 (42.5) |
| No | 30 (20.98) | 87 (23.39) | 22 (11.58) | - | 139 (17.38) |
| Unknown | 61 (42.66) | 151 (40.59) | 14 (7.37) | - | 226 (28.25) |

TCGA-GBM is a national program across the United States. Samples were collected from patients with newly diagnosed GBM based on surgical pathology reports and clinical records. The RNA-seq data composed of fragments per kilobase per million mapped reads (FPKM) values was downloaded from The Cancer Genome Atlas (https://www.cancer.gov/). CGGA693 is a multi-center program contributed by 6 hospitals in China. Samples were collected from patients pathologically diagnosed as gliomas and graded based the World Health Organization (WHO) classification criteria. Glioma samples of GSE16011 were collected from the Erasmus University Medical Center tumor archive (*n* = 276) in Netherlands. Patients were diagnosed based on histological diagnosis and WHO classification (2007).

Table S2. Demographics of all GBM samples included.

| **Characteristics** | **TCGA RNAseq** | **TCGA microarray** | **CGGA693** | **GSE4271** | **GSE16011** | **GSE7696** |
| --- | --- | --- | --- | --- | --- | --- |
| **Data type** | RNAseq | Microarray | RNAseq | Microarray | Microarray | Microarray |
| **Number of samples** | 153 | 494 | 249 | 76 | 159 | 80 |
| **Age at diagnosis (years)** | 59.81±13.75 | 57.50±14.67 | 48.82±13.86 | 48.52±12.82 | 54.10±14.02 | 51.02±9.09 |
| **Gender (%)** |  |  |  |  |  |  |
| Male | 102 (66.67) | 302 (61.13) | 147 (59.04) | 52 (68.42) | 108 (67.92) | 59 (73.75) |
| Female | 51 (33.33) | 192 (38.87) | 102 (40.96) | 24 (31.58) | 51 (32.08) | 21 (26.25) |
| **1p/19q (%)** |  |  |  |  |  |  |
| Codeleted | 0 (0) | 2 (0.41) | 13 (5.22) | - | - | - |
| Non-codeleted | 143 (93.46) | 479 (96.96) | 205 (82.33) | - | - | - |
| Unknown | 10 (6.54) | 13 (2.63) | 31 (12.45) | - | - | - |
| **MGMT promoter (%)** |  |  |  |  |  |  |
| Methylated | 50 (32.68) | 150 (30.36) | 106 (42.57) | - | - | 44 (55) |
| Un-methylated | 67 (43.79) | 175 (35.43) | 93 (37.35) | - | - | 34 (42.5) |
| Unknown | 36 (23.53) | 169 (34.21) | 50 (20.08) | - | - | 2 (2.5) |
| **Radiotherapy (%)** |  |  |  |  |  |  |
| Yes | 120 (78.43) | 406 (82.19) | 195 (78.31) | - | - | 80 (100) |
| No | 21 (13.73) | 65 (13.16) | 34 (13.65) | - | - | 0 (0) |
| Unknown | 12 (7.84) | 23 (4.66) | 20 (8.03) | - | - | 0 (0) |
| **Pharmacotherapy (%)** |  |  |  |  |  |  |
| Yes | 55 (35.95) | 174 (35.22) | 201 (80.72) | - | - | 52 (65) |
| No | 31 (20.26) | 112 (22.67) | 29 (11.65) | - | - | 0 (0) |
| Unknown | 67 (43.79) | 208 (42.11) | 19 (7.63) | - | - | 28 (35) |

GSE4271 program contained a total of 260 WHO grade III and IV glioma samples from M.D. Anderson Cancer Center. Samples were followed up for at least 2 years or until death. DNA microarray was performed to identify gene expression. GSE7696 is a joint project of 85 medical centres in 15 countries. Patients were diagnosed as GBM based on WHO criteria among August 2000 to March 2002.

Table S3. The first screen for gene main effects were of prognostic significance. Genes were included in the Cox-ph model on a case-by-case basis, with age as the covariate.

| **Gene** | **TCGA** | | | | **CGGA1** | | | |
| --- | --- | --- | --- | --- | --- | --- | --- | --- |
|  | **beta** | **CI95** | | **p** | **beta** | **CI95** | | **p** |
| DCBLD2 | 0.235 | 1.002 | 1.599 | 0.048 | 0.153 | 1.030 | 1.317 | 0.015 |
| PXN | 0.586 | 1.145 | 2.820 | 0.011 | 0.207 | 1.007 | 1.503 | 0.042 |
| LOXL1 | 0.275 | 1.104 | 1.569 | 0.002 | 0.110 | 1.007 | 1.237 | 0.037 |
| TFAP2A | 0.362 | 1.108 | 1.861 | 0.006 | 0.217 | 1.077 | 1.433 | 0.003 |
| STC1 | 0.206 | 1.035 | 1.458 | 0.018 | 0.102 | 1.007 | 1.218 | 0.036 |
| EN1 | 0.190 | 1.022 | 1.432 | 0.027 | 0.114 | 1.023 | 1.227 | 0.014 |
| CDC42EP3 | 0.325 | 1.019 | 1.879 | 0.038 | 0.268 | 1.037 | 1.647 | 0.024 |
| COL22A1 | 0.287 | 1.122 | 1.583 | 0.001 | 0.098 | 1.002 | 1.213 | 0.045 |
| TMEM38A | -0.255 | 0.613 | 0.980 | 0.033 | -0.184 | 0.724 | 0.956 | 0.009 |
| IPO11 | -1.070 | 0.145 | 0.810 | 0.015 | -0.326 | 0.523 | 0.996 | 0.047 |
| KIAA1671 | -0.452 | 0.421 | 0.963 | 0.032 | -0.258 | 0.599 | 0.996 | 0.047 |

Table S4. The second screen for gene main effects based on the TCGA cohort. Genes with p values less than 0.05 in the first screen were included in the cox-ph model simultaneously, with age as a covariate.

| **Gene** | **beta** | **Hazard Ratio** | **CI95** | | **p** | **Adj.P** |
| --- | --- | --- | --- | --- | --- | --- |
| DCBLD2 | -0.073 | 0.929 | 0.680 | 1.271 | 0.646 | 0.775 |
| PXN | 0.479 | 1.615 | 0.877 | 2.975 | 0.125 | 0.372 |
| LOXL1 | 0.091 | 1.095 | 0.874 | 1.372 | 0.430 | 0.573 |
| TFAP2A | 0.145 | 1.158 | 0.854 | 1.564 | 0.348 | 0.523 |
| STC1 | 0.026 | 1.026 | 0.829 | 1.271 | 0.811 | 0.848 |
| EN1 | 0.104 | 1.109 | 0.914 | 1.347 | 0.294 | 0.504 |
| CDC42EP3 | 0.219 | 1.245 | 0.902 | 1.718 | 0.183 | 0.401 |
| COL22A1 | 0.188 | 1.207 | 0.972 | 1.498 | 0.088 | 0.353 |
| TMEM38A | 0.025 | 1.026 | 0.792 | 1.328 | 0.848 | 0.848 |
| IPO11 | -0.833 | 0.435 | 0.171 | 1.104 | 0.080 | 0.353 |
| KIAA1671 | -0.666 | 0.514 | 0.318 | 0.830 | 0.007 | 0.079 |

Table S5. 13 G×G interactions in the first screen.

| **NO.** | **Gene1** | **Gene2** | **Coef_seq_** | **P_seq_** | **Cindex_seq_** | **Coef_array_** | **P_array_** | **Cindex_array_** |
| --- | --- | --- | --- | --- | --- | --- | --- | --- |
| 1 | SLCO1C1 | NCAPG | 0.594 | 1.54E-4 | 0.597 | 0.068 | 5.59E-2 | 0.540 |
| 2 | LOXL1 | NCAPG | -0.253 | 6.49E-2 | 0.581 | -0.066 | 7.71E-2 | 0.548 |
| 3 | CNGA3 | DHCR24 | 0.162 | 4.11E-2 | 0.578 | 0.061 | 3.91E-2 | 0.528 |
| 4 | OMG | INA | 0.125 | 3.56E-2 | 0.575 | 0.038 | 7.80E-2 | 0.529 |
| 5 | RIT2 | OAS1 | -0.135 | 3.77E-2 | 0.572 | -0.056 | 6.48E-2 | 0.549 |
| 6 | SLC6A1 | MAD2L1 | 0.328 | 2.69E-2 | 0.565 | 0.076 | 4.60E-3 | 0.540 |
| 7 | UBE2S | NRXN1 | 0.254 | 4.28E-2 | 0.561 | 0.104 | 3.23E-2 | 0.557 |
| 8 | C21orf62 | GOLGA8A | 0.166 | 2.87E-2 | 0.560 | 0.064 | 1.50E-2 | 0.524 |
| 9 | CRYAB | GABBR1 | 0.175 | 2.02E-2 | 0.548 | 0.071 | 2.40E-3 | 0.557 |
| 10 | DIRAS2 | ICAM1 | -0.151 | 6.80E-2 | 0.542 | -0.070 | 3.93E-2 | 0.550 |
| 11 | SLC1A2 | FAM189A2 | 0.163 | 3.56E-2 | 0.540 | 0.076 | 3.76E-2 | 0.525 |
| 12 | HOXA5 | MLLT11 | 0.178 | 2.54E-2 | 0.536 | 0.074 | 2.39E-2 | 0.554 |
| 13 | CTNNA2 | MAD2L1 | 0.270 | 3.46E-2 | 0.526 | 0.075 | 7.14E-3 | 0.526 |

Seq: TCGA RNA-seq, Array: TCGA microarray. The cutoff p-value of each GG interactions was set as 0.08.

Table S6. Gene signature associated with T cell function.

| **Type** | **Genes** | **PMID** |
| --- | --- | --- |
| T cell accumulation  (T accum) | **Positive hit gene**: BTG2, CBLB, CCL1, DGKA, DGKZ, EGR2, ENTPD1, F11R, FYN, HIPK2, JUN, NPTXR, SEMA4D, TAP1, TBC1D22A, WNT10B | 24476824 |
|  | **Negative hit gene**: ACAT1, ALDH4A1, AOC2, BDH1, CAP1, CAPZB, CD200, CD70, CDK2AP1, CDKN1B, CKS2, CRABP2, CST3, CTSD, CYTH3, DOK2, E2F8, ECT2, EEA1, ENO3, ETF1, FOXD1, FZD4, GAA, GAS2, GLS2, GPD2, IL18, IL1R2, IL1RL1, IL2RA, IRF4, ITIH5, JAK3, KLRB1, KLRG1, LAG3, LCLAT1, LITAF, LRIG1, LTB, MDH2, MYB, NR4A2, NUSAP1, PDE6D, PFN2, PGLYRP1, POLD4, PON1, PON2, PRKCQ, PTGFRN, PTPN2, RABGAP1L, RASAL1, RBM39, RGS16, RND3, RNF11, ROMO1, RPA2, S100A3, SAT1, SCG2, SERPINB1, SERPINE2, SKI, SMAD3, TANK, TBC1D4, TIE1, TMBIM1, TMEM109, TNFRSF9, TNFSF11, TTC39B, TTN, UBE2T, VAMP5, XDH, ZFPM1 |  |
| T cell exhaustion  (T exhaust) | **Positive hit gene**: ABCB1, ABI3, ADRB2, BCKDHB, BTG1, C1QC, CAMK2N1, CCL4, CCL5, CD69, CPEB1, CTSS, CUEDC1, CYSLTR2, DEDD2, DHX58, DSEL, DUSP1, DUSP26, FOSB, HSPA1A, HSPA1B, IFIT1B, IFIT3, JUN, MACC1, NR4A2, NR4A3, POU6F1, RGS1, RGS16, RGS2, RNF166, RTP4, SAMD3, SFN, SKI, SLC14A1, SPATA20, SPP1, SYCP2L, TF, TMEM88, TNFAIP3, TNN, TRIM15, VPS37B | 26139534 |
|  | **Negative hit gene**: BANF1, BIRC5, BRI3BP, BUB1B, CD2AP, CENPA, CENPH, CHIC2, CISD1, CKAP2, CMC1, DCAF13, FAM111A, FKBP4, FLNB, GINS4, GPR55, HAUS7, HELLS, IMP4, INTS12, IPO9, KIF4A, MCM2, MITD1, MRPL18, MRPL19, MRPL34, MRPS5, MTHFD1, MTHFD1L, MTHFD2, NDUFA6, NEK2, OXCT1, PDK3, PGAM1, PGD, RFC3, RPP30, SAR1B, SBDS, SETD6, SUCLG2, TOE1, YEATS4 |  |
| T cell exhaustion.2  (T exhaust.2) | **Positive hit gene**: ABI3, ACTN2, AGTRAP, ASB2, BCL2, CA2, CASP1, CCL5, CCR5, CD200R1, CD200R1L, CD244, CD96, CDH1, CHN2, CISH, CXCR6, DAPK2, F2R, FCER1G, FGL2, GLP1R, GRINA, GZMK, HCST, HOXA7, ID2, IL2RB, ITGA1, KIF5C, KLRD1, LRRK1, MYADM, MYO1F, MYO6, NEDD9, OSBPL3, PGM2L1, PPM1B, RGS1, RRAS2, RTN4RL1, S100A9,, SPRY2, TXK, TYROBP, USP20, VAMP4, VPS54 | 28514453 |
|  | **Negative hit gene**: ALCAM, ATIC, BIRC5, BTLA, BZW2, C19orf38, CAPG, CCR7, CD200, CD28, CDCA7, CHST2, CXCR4, DPP4, DUSP4, E2F2, EGR2, EMB, EOMES, FBL, FIGNL1, GALK1, GART, HK2, HSPD1, IL18R1, KPNA2, LEF1, MYB, NRN1, PKN3, POU2AF1, PPA1, SATB1, SELL, SLC29A1, SNN, ST6GAL1, TIMM8A, TNFSF11, UHRF1, USP33, ZBTB32 |  |
| Regulatory T cell  (Treg) | **Positive hit gene**: ANKRD12, ANKRD55, ARHGAP20, CCR6, CCR8, CD200R1, CD80, CD83, CORO2A, CXCL10, DUSP4, ECM1, FOXP3, GPR52, GPR83, IFITM3, IFNGR1, IKZF2, IKZF4, IL10, IL18R1, IL1R2, ITGAE, ITGB8, JUN, LRRC32, LY96, MAF, MYO1E, NEB, NT5E, PENK, PHLPP1, PLAGL1, PTPRJ, RLN3, RRAGD, RTP4, SAMD9L, SCIN, SLC14A1, SLC9A7, TNFRSF8, TRAT1, TTC39C, WLS, ZSCAN29 | 23277554 |
|  | **Negative hit gene**: AKR1C3, ANLN, ARHGAP19, AURKB, BUB1, BUB1B, CCNA2, CCNB2, CDCA5, CENPH, CEP55, DAPL1, DEPDC1, E2F8, ESCO2, FANCD2, FBXO5, HMMR, IGFBP4, KIF11, KIF15, KIF18B, KIF20A, KIF2C, KIF4A, MASTL, MKI67, NCAPG, NCAPG2, NEIL3, NUF2, NUSAP1, PLK1, PRC1, PRR11, RACGAP1, RRM2, SHCBP1, SKA3, SPAG5, SPC25, STIL, STMN1, TPX2 |  |
| ICB resistance  (anti-CTLA4) | **Positive hit gene**: CCR2, CCR5, CD180, CD53, CD74, CD84, CLEC12A, CLEC7A, CTSS, CXCL10, CYBB, CYTH4, DPT, FAM81A, FCER1G, FCGR3A, FCGR3B, FOLR2, FSCN1, GXYLT2, HLA-DQA1, HLA-DQB1, HLA-DRB5, IFI16, IFI44, IL7R, IRF4, IRGM, KCNJ13, LPAR4, LYZ, MGLL, MS4A7, OAS2, PDE8A, PLD4, PLXNC1, PSMB9, PTPRC, RTP4, SERPINB2, SLC7A8, SPP1, TGFBI, TYROBP, USP18, XAF1, ZBP1 | 25754329 |
|  | **Negative hit gene**: ADAM12, AGPAT4, ALAS2, BAHCC1, CACNG7, CDHR2, CDHR4, CRLF1, DEF6, EHD3, ERBB3, ETV5, FXYD1, FXYD3, GPC6, HAPLN1, HDAC9, HKDC1, KCNN4, LGI4, LPIN1, LRP11, LRRC4B, MCAM, MDGA2, MEGF10, MGRN1, MID1, MLH3, MRO, NR4A2, ODF3L1, PDE7B, PGBD1, SCRG1, SEMA6A, SLC35F1, SULF2, TCF7L1, TECRL, TMEM59L, TP53INP1, UGT8, USP2, XYLT1, ZMIZ1 |  |
